# Learning to be well in the health workplace: an integrated model

**DOI:** 10.15694/mep.2021.000045.1

**Published:** 2021-02-12

**Authors:** Matthew Links, Marise Lombard, Benjamin C Forster, Grant Phelps, Paula Brough

**Affiliations:** 1 Griffith University School of Medicine; 2 Griffith University; 3 University of Sydney; 4 Deakin University; 5 Griffith University School of Applied Psychology

**Keywords:** learning, wellbeing, resilience, burnout, organizational psychology, Job demand-resources, mindset

## Abstract

This article was migrated. The article was marked as recommended.

**Introduction:** Problems with the well-being of workers in health is a crisis that directly impacts on health care workers themselves and on the quality of care provided. Academic inquiry has utilised a broad diversity of perspectives. There is an urgent need for theory that guides interventions and mediates between the perspectives taken.

**Methods:** An initial model was generated by mapping concepts from a meta-synthesis of systematic reviews of resilience, burnout, well- being and compassion fatigue. An iterative process identifying and critically applying additional literature refined the model.

**Results:** The final model addressed positive /negative; individual/organisational and focal or global perspectives. It was structured on the Job-demands resources model with stressors mediated by cognitive appraisal, and organisational climate. A cycle of learning in practice was identified as the key to adaptation. The relevant educational domains include learning to be, believe, feel, do, Interact and adapt to maximise well-being.

**Discussion:** An integrated, evidence based learning model of well-being in the health workplace has been developed which may act as a guide for both individuals and organisation to maximise well-being. Implications of the model have been discussed.

## Introduction

The wellbeing of health-workers is an issue with an enormous human toll and impacts on the sustainability, quality and safety of health care systems ( Dewa *et al.*, 2014). Supporting the wellbeing of health care workers is one of four key aims for a quality health system ( Bodenheimer and Sinsky, 2014). The literature has taken three major perspectives: (1) clinical, with a focus on individual psychology, mental health problems (depression, suicide, burnout); (2) organisational, occupational health and organisational psychology; (3) educational, where education has been utilised as an intervention.

An educational perspective suggests learning as a solution ( Bastounis *et al.*, 2016; Stahl, Sharplin and Kehrwald, 2018) but the “wellbeing curriculum” has not been well defined. Educational theory is not well integrated into the occupational health and clinical discourses; and theory of workplace stress are not well integrated into educational discourse around wellbeing.

There is an urgent need for a model of wellbeing in the health workplace that mediates between the different discourses and perspectives taken. An integrated model summarises and mediates the scholarship in multiple domains and helps clinical educators and health managers implement solutions.

## Aim

Develop a learning model of wellbeing that builds on existing models of health and learning that is fit for purpose to guide interventions.

## Methods

**Figure 1.**
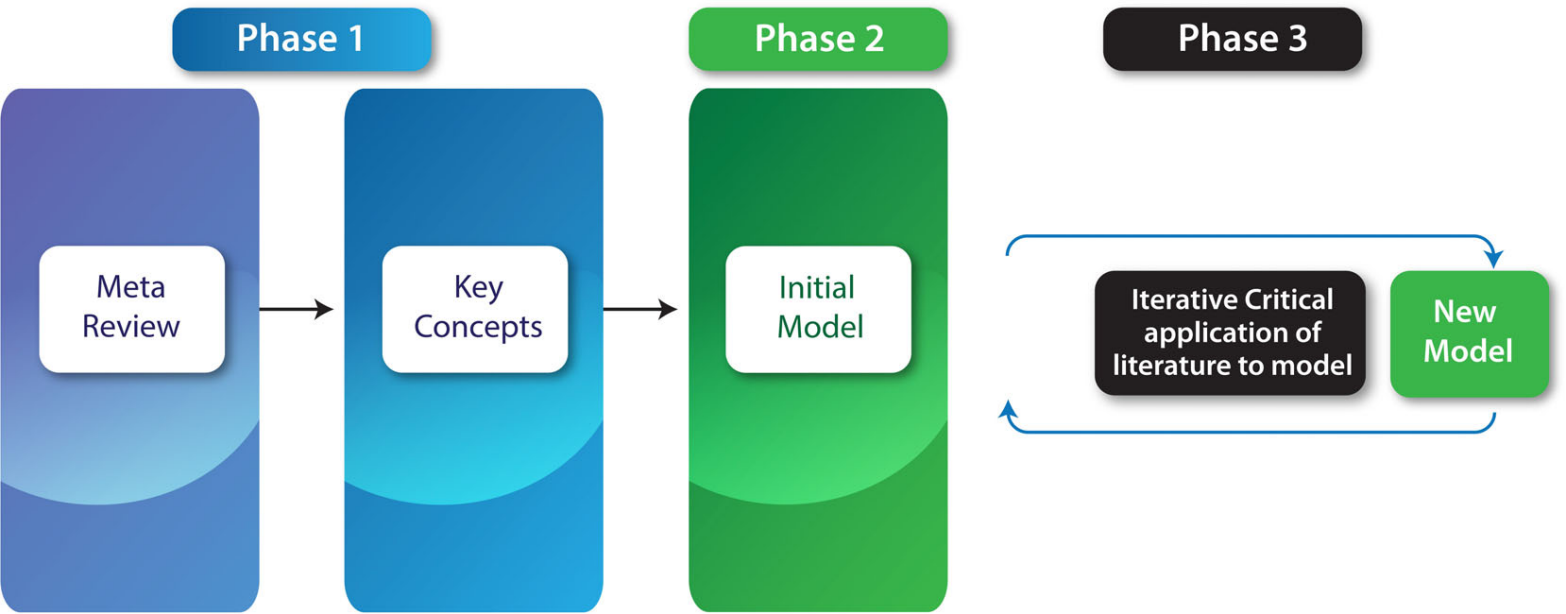
Summary of methods: Phase 1 identification of the literature. Phase 2 generation of an initial model; Phase 3 iterative improvement of the model.

The methods used a systematic review to identify a starting point in the literature, but subsequent questions were explored non-systematically to allow an iterative and expansive approach to the questions asked. This process was guided by the SRQR standards for qualitative research ( O’Brien *et al.*, 2014). The literature analysis involved considerations of underlying paradigms and assumptions and drew upon the RAMESES standards (Realist And MEta-narrative Evidence Syntheses: Evolving Standard) with principles of pragmatism, pluralism, historicity, contestation, reflexivity and peer review ( Wong *et al.*, 2013). The methods are summarised in Figure 1.

Phase 1 was a review of underlying concepts and perspectives. Given an extensive existing literature of systematic reviews, a meta-review was performed of the published systematic reviews of resilience and burnout in health care workers. This review was then expanded to include other concepts such a compassion fatigue. This enabled identification of existing models; key concepts; and perspectives. The initial (primary) literature search was conducted by two authors (ML1 and ML2) using the Search strategy:  Psychological resilience, or compassion fatigue or burnout, professional [MESH term or Keyword] AND systematic review [Title]). Databases search included PubMed, psychoinfo and CINAHL.

Papers were selected for inclusion using the selection criteria that: (1) it was empirical data; (2) the focus of the study was well-being or resilience; (3) that the subjects were health-care  workers; (4) that the challenge for resilience or well-being was working in the health-system  (not a disaster or other threat); and (5) that the language was English. 

Given the interest in including broader concepts of wellbeing a search for the broader term of wellbeing and health care providers was conducted in PubMed. The large number of papers identified were of low relevance so a process of screening till saturation was adopted. This entailed using the PubMed sort by relevance function (Best Match) ( Fiorini *et al.*, 2018) and screening the results according to the selection criteria. This process was continued until 100 consecutive studies were screened without an eligible study being identified.  

A variety of quality frameworks were considered to assess the quality of papers identified. However, these prioritized methodological and procedural quality of systematic reviews rather than the quality of the concepts ( Long and Godfrey, 2004; Kitto, Chesters and Grbich, 2008; Kuper, Lingard and Levinson, 2008; Pace *et al.*, 2012). None of the existing tools were fit for purpose. For the purpose of this analysis the critical question was not whether the review was conducted rigorously but whether the conceptualisation of the problem added to the existing model. This judgement by the authors was part of the iterative process of model generation.  

Themes were identified and recorded in a excel spreadsheet by two authors (ML1 and BF). Identified literature was recorded in a Zotero database.   

Phase 2 was generation by one author (ML1) of an initial model through mind-mapping of concepts onto the IPO model and subsequent modification by consensus of the research group. Mind-mapping was chosen as an open ended, visual way of situating identified concepts within an initial framework ( Crowe and Sheppard, 2012). 

Phase 3 was an iterative process of model refinement by examining the proposed model from different perspectives. An underexamined perspective was identified during the previous phase of inquiry. Additional literature was identified through focused searches, forward and backward snowballing and from personal databases. Consideration was given beyond the health field and the initial focus on systematic reviews. Theoretical perspectives trusted by relevant communities of practice were prioritised. Trust was assessed by the utilisation of models in subsequent research and by expert assessment of peer opinion. The literature database was supplemented by additional searches and the model evaluated according to the following criteria: (1) Is the model comprehensive; can it be simplified; Is it a practical; and what other perspectives need to be addressed?  

Rigour was addressed by careful consideration of researcher expertise and characteristics with recruitment of additional authors to provide alternative perspectives. The final research group included expertise in medicine, education, nursing, health management and organisational psychology. Reflexivity was promoted through an online reflective log and an iterative process of critical evaluation and consensus through teleconference, face to face meetings and circulation of drafts by email. Trustworthiness of the final result was enhanced by prioritisation of trusted existing models within the model building process, thus building on existing critical opinion. 

## Results

### Phase 1: Identifying the literature

A total of 146 studies were identified by the original search strategy in PUBMED examining reviews of psychological resilience, compassion fatigue and professional burnout. The search was then expanded to include CINAHL (n=96) and Psychinfo (n=41). An initial theme identified was the difference between the individual psychological and organisational perspective, so the search was expanded to include organisational climate and occupational health (n=55). Papers that did not deal with health workers, were opinion pieces or were not focused on the wellbeing of health workers were excluded, producing 136 papers for analysis. 

**Figure 2.**
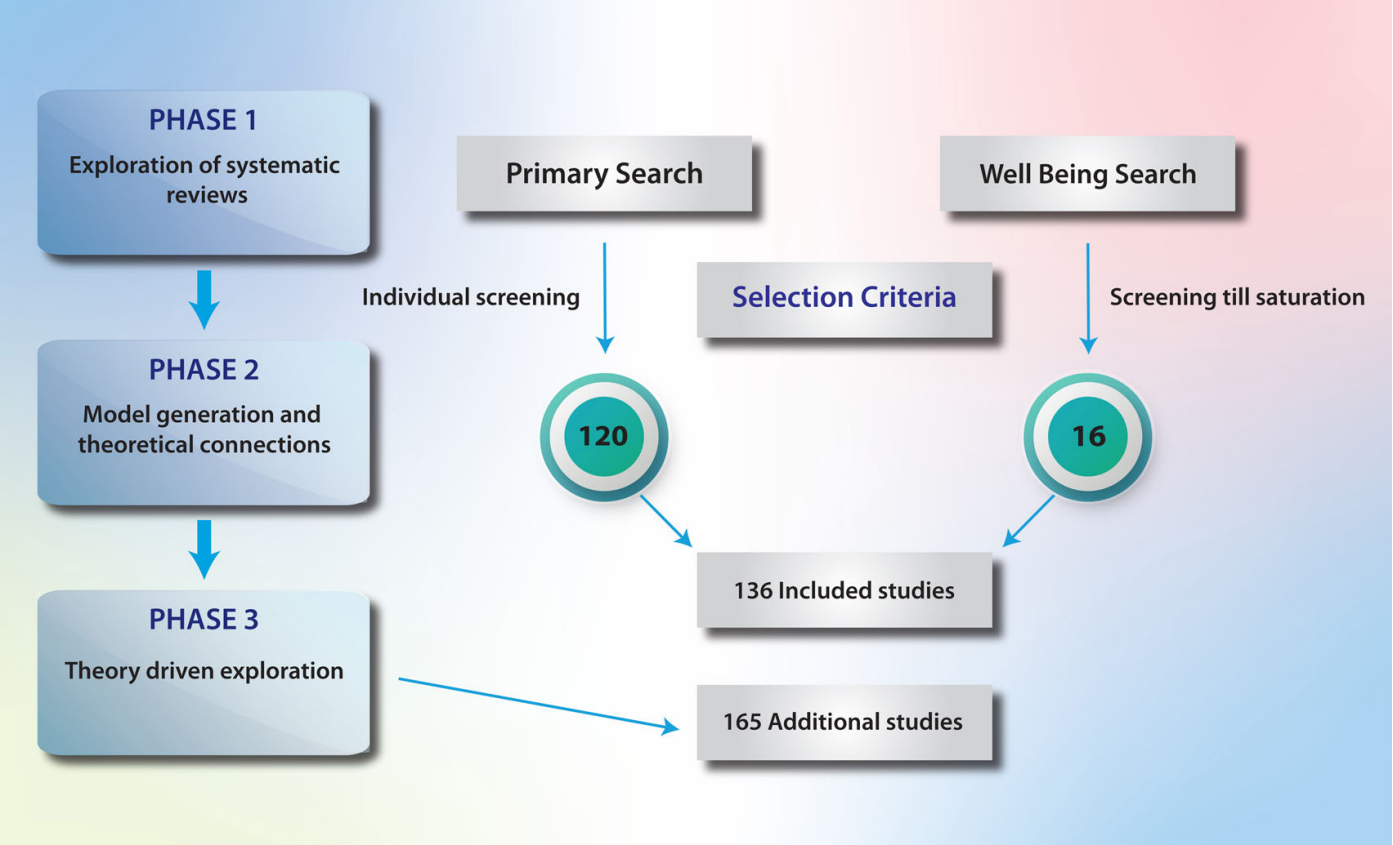
Results of the literature search.

The identification of literature in Phase 1 is summarised in Figure 2

**Figure 3.**
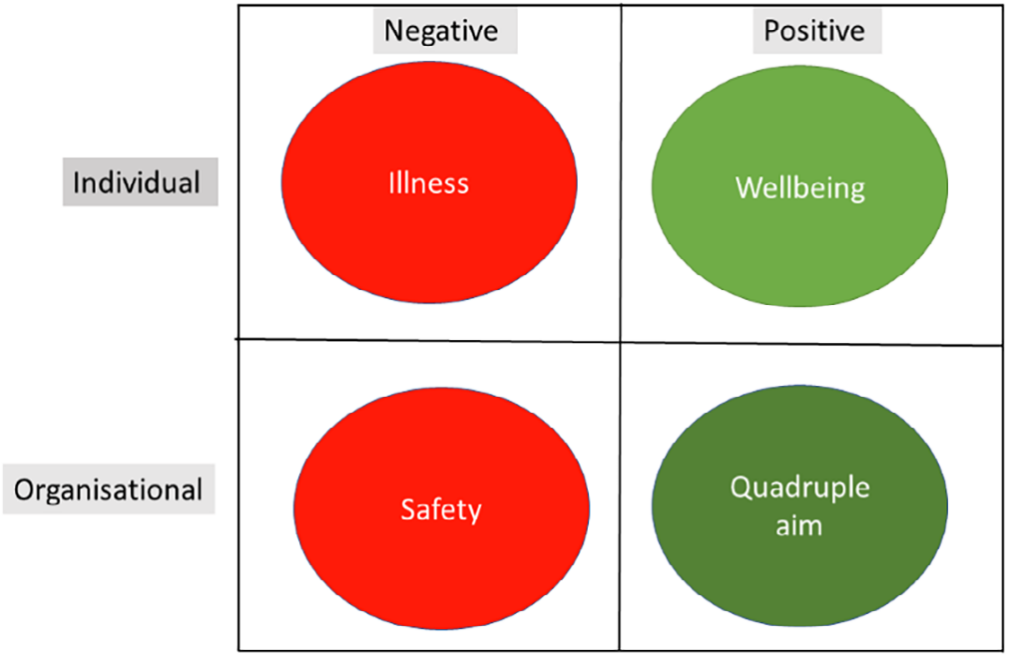
Variations in perspectives taken. Global perspectives were divided into a matrix of positive versus negative and individual versus organisational.

Significant variations in the perspectives taken were identified. Although these perspectives are represented as black and white distinctions, it is acknowledged that this is a simplification and that there are continuums in perspective and significant overlaps. Perspective were classified as: Individual versus organisational; positive versus negative; and global versus focused. Burnout, as an example, is an individual negative and relatively global perspective, contrasting to the positive perspective of resilience and compared to more focal constructs of compassion fatigue or existential burnout/distress. These difference in perspective are summarised in Figure 3. 

Papers were classified into major and minor themes. The major perspective taken was individual rather than organisational. Major themes were descriptions versus interventions:  Minor themes were: existential issues (n=10); consequences (n=6) and biological factors (2). A historical trajectory was identified in the questions being asked and the theory and terminology utilised. A critical approach identified the influence of power within these transitions as there have been strong feelings on the part of many health workers that an individual focus has been a way of attributing blame and avoiding organisational responsibility ( Chamorro-Premuzic and Lusk, 2017; Aubusson, K, 2017). 

A major variation was that of focus. The Input-processes output model was identified as a useful organising structure. Using this system focus was on: (1) inputs (job demands, resources etc); (2) processes that link inputs and outputs; (3) outputs (resilience burnout, wellbeing, quality and safety of care etc); and (4) environment (consideration of the environment that these processes occurred in).

The terminology used described a historical trajectory from workplace stress to outcomes of stress: burnout, resilience and wellbeing ( Eckleberry-Hunt *et al.*, 2009). A very broad range of overlapping terminology was identified with distinctions between concepts often problematical ( Southwick *et al.*, 2014; Bianchi, Schonfeld and Laurent, 2015; Litz and Kerig, 2019). Burnout, resilience, workplace stress, moral distress, secondary trauma, compassion fatigue, and workplace health were all identified as relevant terms within the literature. Differences in the perspective take were partly accounted for by differences between a positive (resilience) or negative (burnout) perspective and partly by variations in scope from a focused outcome (absenteeism or moral distress) to holistic concept such as wellbeing. 

There has been a historical trajectory in the theoretical perspective taken. Initial work was based on application of behavioural models of stress to the workplace with a focus on the psychological processing of stress and the identification of important concepts such as self-efficacy, autonomy and baseline personality traits. Important theoretical constructs were Lazarus and Folkman’s transactional model which highlighted that the response to stress is a **process** which is mediated by a process of cognitive appraisal of the work environment ( Lazarus and Folkman, 1987).   

Early work focused on precise definition of terms and tools for measurement of well-defined **outputs** of workplace stress such as the development of the Maslach burnout inventory ( Schaufeli, Maslach and Marek, 2017), asking questions around prevalence in different communities or professional groups ( IsHak *et al.*, 2013; Adriaenssens, De Gucht and Maes, 2015; Chuang *et al.*, 2016; Dimou, Eckelbarger and Riall, 2016). Important concepts were the construction of burnout as a response to stress with dimensions of exhaustion, disengagement and lack of accomplishment. The question was often -what is the prevalence in my “tribe”: physician burnout or burnout in nurses rather than a focus on teams in the same work environment. The introduction of work hour limits in Europe and the USA led to an evaluation of the impact of changing this measure of job demand ( Martini, Arfken and Balon, 2006; Landrigan *et al.*, 2008). 

The accumulating literature on prevalence across disciplines has led to the concept of a crisis and increasing calls for action highlighted by high profile and personal examples. The COVID-19 pandemic has highlighted the implications of the physical and emotional health of health workers in a dramatic way. 

A major focus, particularly of early models was identification on the relative importance and interactions between various **inputs** into wellbeing. This was reflected in the demonstration of the importance of imbalance models such as job demands and resources ( Bakker and Demerouti, 2007), control ( Demerouti *et al.*, 2001) or rewards ( Basińska and Wilczek-Rużyczka, 2013; Morgan, Dill and Kalleberg, 2013). The question was often: what are the important determinants of (stress, burnout, resilience) in this context? Study of the outputs of workplace stress have moved from a negative (burnout) to a positive perspective (resilience) to a more holistic perspective (wellbeing). These shifts have coincided with a parallel discourse around how we define health ( Huber *et al.*, 2011). This is reflected in a move from an absence of illness-based definition, to a more holistic assessment of human needs. Influential perspectives include Maslow’s self-actualisation theory ( Maslow, 1970), and Sens capability approach ( Sen, 1993). 

Subsequently the focus has moved to **interventions**, particularly psychological interventions conceived as “stress management”( Reynolds, Taylor and Shapiro, 1993; Bragard *et al.*, 2010); from cognitive behavioural therapy ( Gardner *et al.*, 2005) to massage ( Brennan and DeBate, 2006) and aroma therapy ( Hansen, Hansen and Ringdal, 2006). More recently mindfulness-based stress reduction has been a dominant method ( Goodman and Schorling, 2012; Luken and Sammons, 2016; Daya and Hearn, 2018), and there has been a more dynamic approach focused on recovery, and a more holistic consideration of environment to include home and (work-life balance). 

Consideration of workplace stress as a **process** highlighted the mediating role of specific behaviours between perception and outcomes. These behaviours included practices of clinical care, selfcare and learning. Learning was identified as the key to adoption of adaptive or mal-adaptive behaviours leading to positive or negative outcomes. 

The organisational perspective reflects a fundamental recognition about the inadequacy of an individual perspective and the importance of the **environment**. This also has undergone a historical trajectory that has moved from a focus definition and measurement of summative concepts such as organisational culture and climate towards a more differentiated approach to construct climate in multiple ways e.g. a safety climate or learning climate. The organisational perspective has also moved towards a more dynamic consideration of how adaptation occurs, with a greater focus on recovery, as well as a more holistic approach to the way that work interacts with home (work-life balance or integration). 

The key concepts and theoretical frameworks identified, definitions and associated references are outlined in Supplementary File 1. 

### Phase 2: Identification of initial model assumptions 

**Figure 4.**
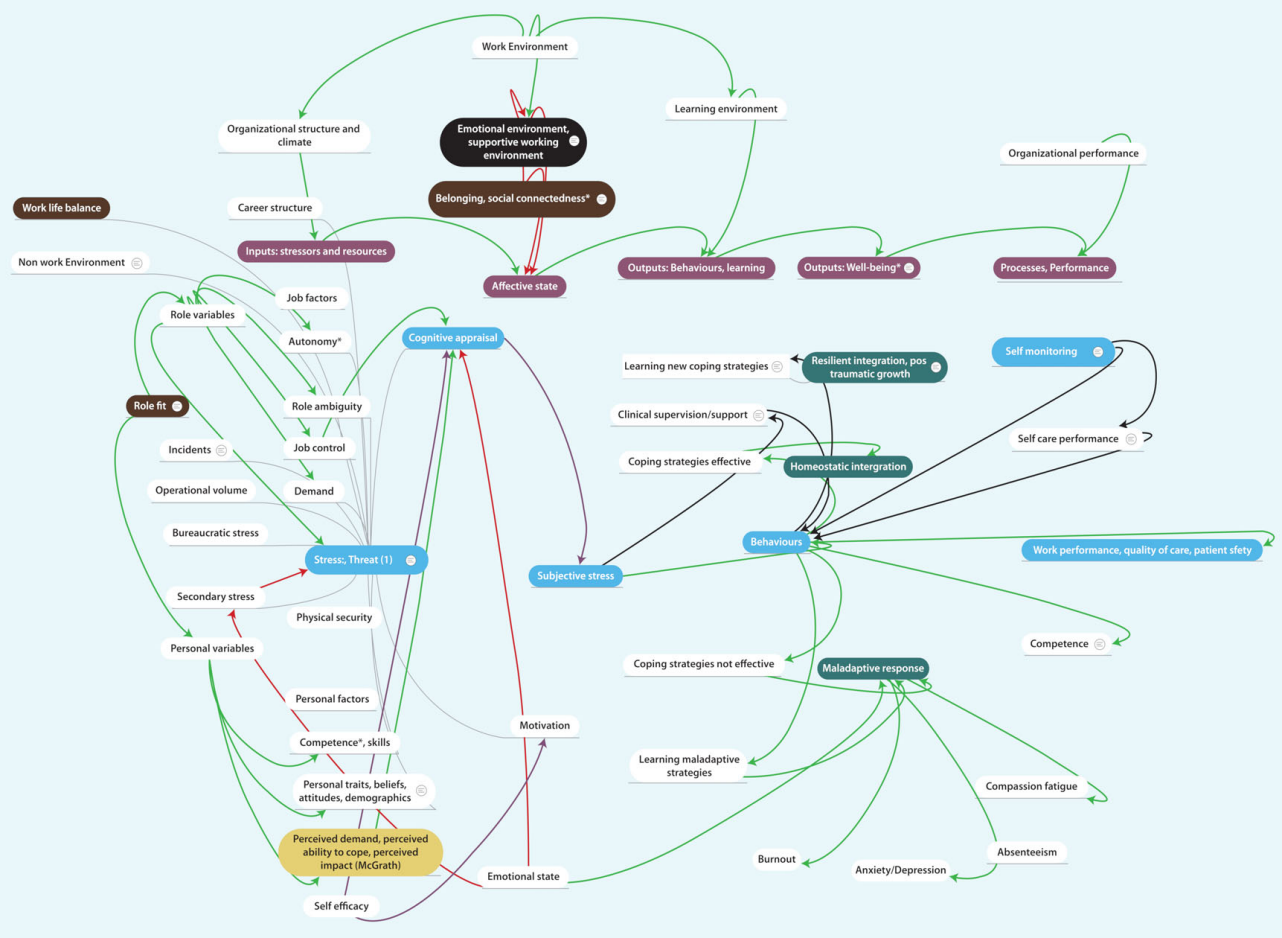
The initial mapping of concepts from phase 1 onto a mind map. Concepts identified in the literature review are mapped onto this framework.

These considerations lead to the adoption of the input process and outcomes model (IPO) as a basic framework to provide a trusted organising conceptual framework. The foundational model for stress-response has been the transactional stress model, so this was fitted to an IPO model. “Objective” inputs into the workplace were identified as stressors/threats, outputs of the system divided into wellbeing and performance (individual or system). The mediating processes are: (1) cognitive appraisal in identifying the perceived stressors; and (2) the identification of specific behaviours (clinical practices, self-care behaviours, etc). The adaptation to workplace stress occurs within a specific work climate or organisational environment. The IPO model, Lazarus workplace transactional stress model and organisational climate literatures, were therefore identified as key elements for the initial model. Characteristics of the initial model are given in Figure 4 and published online at Mindmeister .

### Phase 3 Iterative improvement 

#### Improving comprehensiveness 

The model was revised by targeted exploration of theory around the concepts outlined, expanding searches, where necessary beyond systematic reviews, beyond health and by recruiting new theoretical perspectives. McGrath generalised the role of perception in stress to include perceived demand, perceived ability to cope, perceived impact ( McGrath *et al.*, 1970). Inputs of stress have been summarised by Maslach’s six areas of  worklife ( Leiter and Maslach, 1999) and the concept of wellbeing elaborated by Warr’s vitamin theory ( Warr, 2008). Concepts of recovery were strengthened by the stressor-detachment model ( Sonnentag and Fritz, 2015). The issue of environment was broadened to differentiate between different aspects of environment such as the climate of psychological safety ( Dollard and Bakker, 2010) and learning ( Lases *et al.*, 2019) which impact in different ways. The coherence of these concepts with the initial model increased its trustworthiness. 

#### Reducing complexity 

Subsequent improvement of the model focused on reducing complexity and synthesis of overlapping concepts within the existing model. Much of the complexity to the model comes from integrating organisational and individual perspectives so the model was simplified from a comprehensive model (simultaneously dealing with individual and organisation levels) to a multi-level model (able to be applied to each level). Identification of learning as a key process led to consideration of learning at either as individual learning ( Sherwood and Zomorodi, 2014). Inputs were consolidated using the six areas of worklife ( Leiter and Maslach, 1999) and the literature on mindsets; ( Sherwood and Zomorodi, 2014, Yeager and Dweck, 2012; Ben-Avi, Toker and Heller, 2018) was introduced as both an input to the system and a potential learning focus. The concept of a mindset or schema allowed collation of multiple beliefs about stress, clinical practice and improvement in ways that impact on the response to stress. Thus, a growth mindset, where the ability to cope with stress is not considered a fixed trait, promotes resilience ( Yeager and Dweck, 2012). Promoting and teaching such a mindset becomes a critical strategic priority. 

Outputs were consolidated into those related to wellbeing and performance with a continuum of outcomes between negative (burnout, clinical error) and positive (resilience wellbeing and high-quality care). 

#### Identification of additional perspectives 

Two areas were identified as under-theorised in the initial model. The first was the relationship of the proposed model to the Job demand-resources model which was identified as a highly trusted model in the existing literature. The second area was that of learning which had a role in many of the processes identified and potential interventions. These areas were selected for further iterations of inquiry. 

#### Re-appraisal against existing models (JD-R) and focus on learning 

The major difference from the simple presentation of the JD-R model was the highlighting of the difference between appraised job demands/resources and the environmental job demands. Consideration of learning in practice to be a critical mediator of wellbeing raises the question as to what drives this cycle? We drew on the JD-R models conceptualisation of the key role of energy and motivation to highlight these as drivers. The major difference between this model and other models, was the focus on learning in the workplace. This raised the question as to what is learnt. We therefore consolidated relevant learning domains as increasing circles of focus from the inner self to the iteration with others. Thus, drawing on professional identity, the practitioner learns to be a doctor or a nurse, reflecting the critical existential domains of burnout and wellbeing. Drawing on the literature on cognitive appraisal and mindsets there are important beliefs which are both inputs into the system and learnt outcomes. Beliefs such as self-efficacy, a growth mindset - where obstacles are opportunities for learning and beliefs that they system is supportive of you - or not. A further internal aspect of wellbeing related to the affective domain of learning and the learning of skills in emotional self-regulation and self-care. 

**Figure 5.**
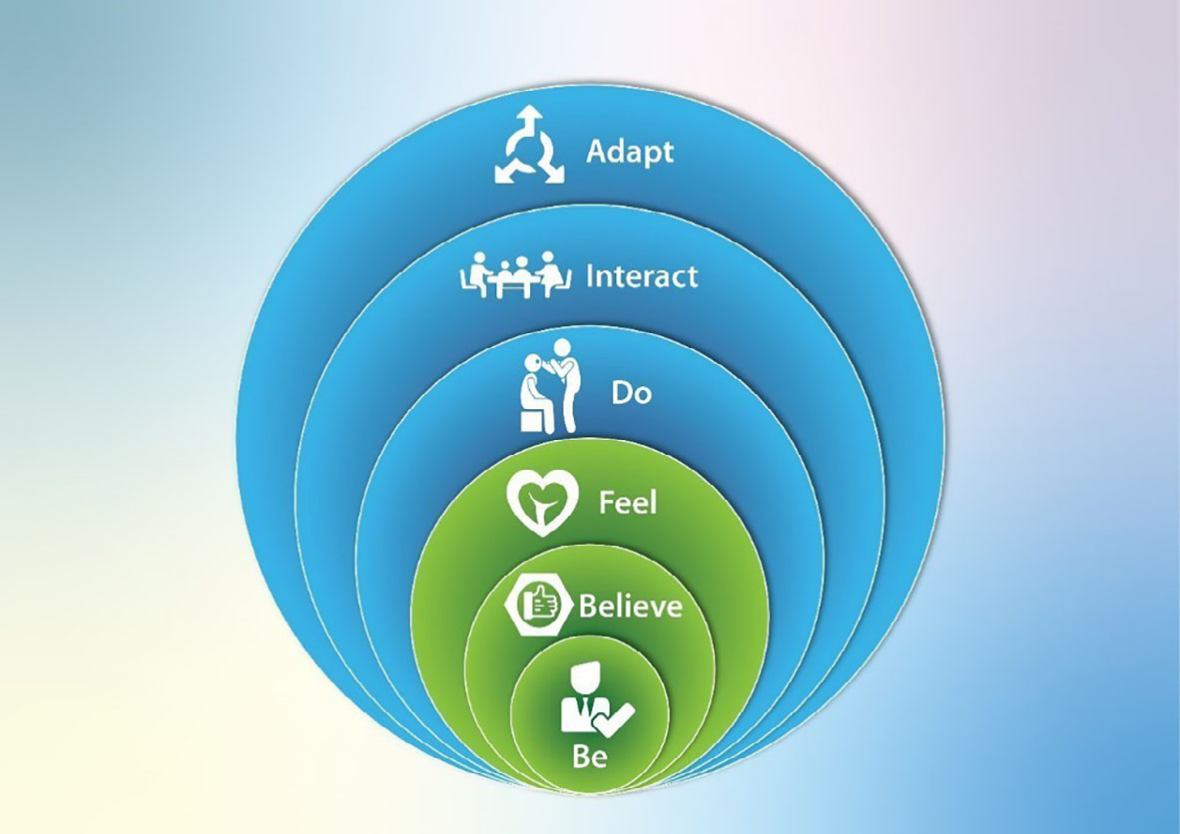
Domains of learning relevant to stress and wellbeing. The domains of learning are represented as moving in increasing circles from the personal to the social.

A critical domain of learning is what practitioners do, of practice. This includes learning to communicate better, learning to insert an airway efficiently while minimising risk of self-contamination, learning to set aside time for reflection and study. The team-based nature of contemporary health practice does require moving beyond the individual perspective and here the practitioner needs to learn how to interact. Skills in teamwork, dealing with interpersonal conflict, asking for help etc, are important skills for avoiding burnout, thriving and delivering high quality care. The final level of learning moves from a static perspective to a dynamic one and that is learning to adapt. Learning to learn, learning how to respond to adverse events, planning self-care when strategies are not working are necessary in a dynamic model. The domains of what is learnt are represented in Figure 5. 

The final model therefore makes explicit that the wellbeing can be considered at different levels as the wellbeing of the individual, the organisation or the society; and that each level goes through a process of appraisal of demands and resources, which mediates stress. The main process is an iterative process of learning and adaptation of behaviours. The drivers of this process are motivation and energy. These produce outputs of wellbeing and performance. Each locus of concern interacts with its own environment which moves from the micro to macro approach. The environment however can be deconstructed into three main domains- the “objective” environment of physical resources and demands, the appraisal environment which influences how an individual or organisation views the situation and a learning environment which influence show behaviours can adapt. 

**Figure 6.**
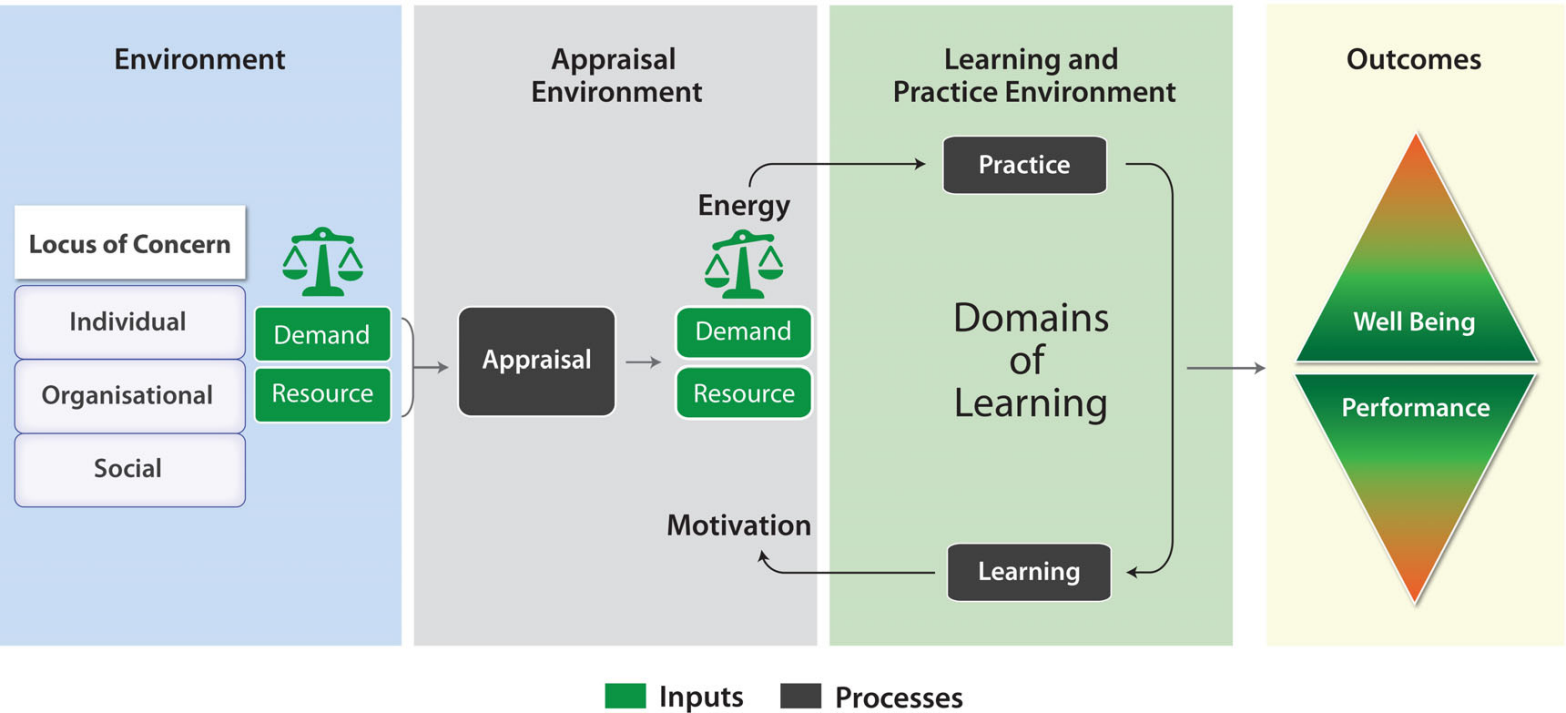
A model of learning to be well in the workplace in response to stress.

The issue of what learns and what adaptation occurs can be viewed similarly as circles moving from critical identity questions of “being”, beliefs, emotional response and practice to questions of interaction and adaptation. The final model is represented in Figure 6. 

## Discussion

This evidence based, multi-perspectival and pragmatic model developed has been developed through a reflexive iterative process drawing on scholarship from multiple discourses. It avoids potential problems associated with choice of either an individual/personal, positive/negative or focused or holistic perspectives associated with existing models of burnout, resilience or workplace stress. The dynamic nature of the model as a cyclical process of experience, learning and adaptation is particularly suited to the implementation of interventions designed to improve wellbeing in the workplace.

Implementation science suggests that solutions to wicked problems need to be tailored to individual contexts and multi-level. Single interventions are likely to have small effects. The first implication of this is that there is a need to understand context and the need for a framework to address what questions are relevant. When assessing the needs, barriers, or enablers in a given context the first step is to make sure that the map covers the whole ground considered relevant by scholarship in this area ( Jacobs, Weiner and Bunger, 2014).

The adoption of a dynamic learning in practice model implies that at both an individual and organisational level there is a need for assessment of outcomes from wellbeing and performance perspectives. This data is then available to drive improvements and to make decisions in a learning organisation and individual. This data needs to include a contextual understanding of where the problems and potential improvements from a holistic viewpoint that considers the physical environment, personal and cultural factors impacting on appraisal, as well as the ability to learn, implement improvements and adapt.

The inputs to the system are dominated by demands and resources and the evidence suggest that most of the variance in wellbeing related to organisational factors not individual ones ( Williams *et al.*, 2002). Any plan needs to consider the quadruple aim where the wellbeing of staff and the contribution to a sustainable system is balanced against considerations of volume and quality. There is limited capacity to reduce demand or increase resources in health thus the promotion and teaching of efficiency in practices are strategic learning objectives.

The process of appraisal is critically impacted by mindsets that are influenced by personal learning and organisational cultures. Teaching modelling and promoting growth, improvement and systems thinking mindsets are methods of promoting resilience, improving performance and reducing stress. The acknowledgement of the impact of emotions and social support on stress, learning and performance highlights the need for a plan that addresses emotional and social needs as well as cognitive ones

The importance of drivers of energy and motivation as drivers of learning in practice highlights the need for reasonable workhours, promotion of recovery strategy to enhance energy and a culture that explicitly addresses motivation. The importance of learning environment highlights the critical role of clinical supervision both in creating opportunities to learn, safely evaluating performance, and providing support in a challenging environment.

The teasing out of the different elements to be learnt as learning to: be, believe, feel, do, interact and adapt is the first step in developing a curriculum of wellbeing. Any educational programs to enhance wellbeing should consider what combinations of these issues are most important in the current context. Teaching mindfulness is an evidence-based strategy but the potential drivers of learning to be well are much broader than any single strategy.

Organisational learning reprises these concerns but one difference at an organisational level is the importance of organisational structure and positional leadership. Organisational learning requires leadership of change ( Senge, 1996) and a climate that promotes continuous learning ( Eldor and Harpaz, 2016; Lases *et al.*, 2019). Individual Leadership styles impact on satisfaction and wellbeing  ( Skakon *et al.*, 2010; Cummings *et al.*, 2018) and there is a need to support leaders’ own well- being and learning (84).

The highlighting of the different aspects of the environment that are relevant to wellbeing emphasises the role of the organisation in establishing a climate that manages resources, promotes positive appraisals and promotes learning. At an organisational level the relevant environment is the broader social environment and prevailing culture around valuing the contribution of healthcare workers, prioritising wellbeing against productivity and investing in learning. Key implications of the model are given in Table 1.

**Table 1.** Key implications of the model

Well-being needs to be a priority
Both inputs (stressors) and processes (adaptation) are important
Data on both well-being and performance are needed to drive improvement
Motivation and energy are required
Growth, improvements and systems thinking mindsets enable adaptive behaviours
Learning to be well involves being, believing, feeling, doing, interacting and adapting
Doing/practicing more efficiently is a win for both well-being and performance
Organisational leadership is critical

The COVID-19 pandemic highlights many of these issues and has brought the wellbeing of health workers to become front page news. Public health measures to manage demand and identify resources have been key issues. However, resilience becomes a secondary issue in the absence of adequate supplies of personal protective equipment. The pandemic has required enormous changes in practice for individuals and organisations which have required learning, adaptation and dissemination. The appraisal of the situation has been powerfully influenced by issues of meaning, community support and the framing of this work as justifiably heroic.

Limitations of the proposed model relate to balancing simplicity with utility. It is not the only potential model and the benefits over a simpler, or more complex model, are yet unproven. The application depends on how job demands and job resources are conceived. Furthermore, consideration of factors outside the workplace depends on whether they are included as an individual resource or demand. The model is explicitly a learning model and therefore does not take into account fixed personality traits, which may be relevant for other approaches such as selection into a profession.

Another limitation of the multilayer model, separating individual and organisational models, is that it does not emphasise interactions between the layers such as in the job-fit model ( Chatman, 1989). The wellbeing of individual healthcare staff can be considered both a result of and a contributor to, the organisation’s performance and its way of working ( Dickson-Swift *et al.*, 2014).

The model does lead to some empirically testable hypotheses: are the environment, appraisal environment and learning environment separate entities and how do existing tools address these conceptual differences?  What level should educational interventions be aimed at along the continuum from being to adaptation? Can this be assessed and tailored to individual workplaces or individuals? How does this framework support the development of individual or organisational wellbeing plans?

## Conclusion

Paracelsus is credited with the statement that all models are wrong, but some models are useful. The strength of this model is that it integrates multiple discourses about health, wellbeing, burnout, occupational health and education, towards a model that is potentially useful in guiding the scope of interventions. Organisations need a conceptual model and a methodology for improving the wellbeing at both individual and organisational levels. The wellbeing of the health workforce, as well as the quality, safety and sustainability of the health system, demand that we get this right.

## Take Home Messages

• Wellbeing in the health workplace is critical to the quality of care provided, the sustainability of the workforce and to individual practicitioners.
• Job demands, resources, perceptions, work evironment and adaptations all contribute to well being.
• Both organisations and individuals can learn welllbeing skills and practices.
• A curriculum of well being involves learning to be, feel, believe, do, interact and adapt in ways that are helpful.

## Notes On Contributors

**Matthew Links** is a medical oncologist and educator with appointments as Professor at Griffith University Medical School and Griffith Institute of Educational Research. ORCID ID: https://orcid.org/0000-0003-3779-3705

**Marise Lombard** is Lecturer in the School of medicine at Griffith University with degress in social science, nursing and midwifery and a Doctorate in Medical Education. ORCID ID: https://orcid.org/0000-0002-7883-4262

**Benjamin C. Forster** is a medical oncologist, palliative care physician and Clinical Senior Lecturer in the School of Medicine University of Sydney. ORCID ID: https://orcid.org/0000-0001-9356-8639

**Grant Phelps** is a Gastroeneterologist, Medical Administrator and Associate Professor of Medical Leadership at Deakin University.

**Paula Brough** is Professor in the School of Applied Psychology, Griffith University. ORCID ID: https://orcid.org/0000-0002-0374-0026
