# Supplementary material for: Learning to be well in the health workplace: an integrated model
Source: MedEdPublish (2016). 2021 Feb 12;10:45. [Version 1] doi: 10.15694/mep.2021.000045.1 (PMC10939621; doi:10.15694/mep.2021.000045.1)
Supplement: Peer review history

# Reviewer report: Reviewer response for version 1

**DOI:** 10.21956/mep.18850.r26872

**Published:** 2021-02-18

**Authors:** Balakrishnan Nair

**Affiliations:** 1 Centre for Medical Professional Development and University of Newcastle

This review has been migrated. The reviewer awarded 4 stars out of 5 This is a very timely article. The Covid 19 epidemic highlighted may stressors for health care professionals. Most of the academic articles are on how to look after patients. But who looks after the health care professionals ? As the authors point out there is compassion fatigue and the individual clinician and the system should work together to look after the well being of the clinician. This in turn will make the patients and the system much better .

Reviewer Expertise:

NA

No decision status is available

# Reviewer report: Reviewer response for version 1

**DOI:** 10.21956/mep.18850.r26871

**Published:** 2021-02-15

**Authors:** BALAJI ARUMUGAM

**Affiliations:** 1 TAGORE MEDICAL COLLEGE AND HOSPITAL

This review has been migrated. The reviewer awarded 4 stars out of 5 This study was done with the background of understanding the well being of health-workers is an issue and highlighted the urgent need for a model of well being in the health workplace. The authors have done a beautiful integrated model with the help of ROL on 136 studies examining reviews of psychological resilience, compassion fatigue and professional burnout. They succeeded with the model demonstrating in Figure – 5 – Showing Be, Believe, Feel, Do, Interact, Adapt - The domains of learning are represented as moving in increasing circles from the personal to the social. A model of learning to be well in the workplace in response to stress with the final model focusing on Physical environment, Appraisal environment and Learning environment. The need of the hour especially in the midst of COVID pandemic the health workers are under tremendous stress and burn out has became common among all the health care workers. The individual level model was well explained but the organisational level adaptations and challenges towards the health care workers was given lesser importance in this review. ANYWAY CONGRATS TO THE AUTHORS FOR THE GOOD REVIEW ARTICLE.

Reviewer Expertise:

NA

No decision status is available

# Reviewer report: Reviewer response for version 1

**DOI:** 10.21956/mep.18850.r26870

**Published:** 2021-02-14

**Authors:** P Ravi Shankar

**Affiliations:** 1 American International Medical University

This review has been migrated. The reviewer awarded 4 stars out of 5 Thank you for the invitation to review this interesting manuscript. The authors try to develop an integrated model of well-being in the health workplace. The methodology is well described though some of the technics used during the search and data synthesis may require more explanation. I am interested in knowing more about forward and backward snowballing. Taking the perspective of a general reader of the journal some of the concepts and discussions may need greater explanation. Supplementary file 1 is useful and the article proceeds from an assumption that most readers will be familiar with the concepts described. This may or may not be true for these interdisciplinary areas. Some basic information has been provided in the supplementary file. Complexity, systems thinking, resilience, well being are becoming important, and as the authors highlight the ongoing coronavirus pandemic has focused attention on these issues. The article may require repeated reading and careful reflection. The charts and figures are useful to better understand this complex topic.

Reviewer Expertise:

NA

No decision status is available
